# Optimizing G6PD testing for *Plasmodium vivax* case management and beyond: why sex, counseling, and community engagement matter

**DOI:** 10.12688/wellcomeopenres.15700.2

**Published:** 2020-08-25

**Authors:** Cindy S Chu, Germana Bancone, Maureen Kelley, Nicole Advani, Gonzalo J Domingo, Eva M Cutiongo-de la Paz, Nicole van der Merwe, Jessica Cohen, Emily Gerth-Guyette

**Affiliations:** 1 Shoklo Malaria Research Unit, Mahidol Oxford Tropical Medicine Research Unit, Faculty of Tropical Medicine, Mahidol University, Mae Sot, Thailand; 2 Centre for Tropical Medicine and Global Health, Nuffield Department of Medicine, University of Oxford, Oxford, UK; 3 The Ethox Centre and Wellcome Centre for Ethics and Humanities, Nuffield Department of Population Health, University of Oxford, Oxford, UK; 4 PATH, Seattle, Washington, USA; 5 Institute of Human Genetics, National Institutes of Health, University of the Philippines Manila, Manila, Philippines; 6 Philippine Genome Center, University of the Philippines System, Quezon City, Philippines; 7 Division of Chemical Pathology, Department of Pathology, Faculty of Medicine and Health Sciences, Stellenbosch University, Tygerberg Academic Hospital, Cape Town, South Africa

**Keywords:** G6PD deficiency, Plasmodium vivax, neonatal hyperbilirubinaemia, gender, sex, disparity, G6PD testing, primaquine, tafenoquine, genetic counselling, haemolysis, G6PD heterozygous females

## Abstract

Safe access to the most effective treatment options for *Plasmodium vivax* malaria are limited by the absence of accurate point-of-care testing to detect glucose-6-phosphate dehydrogenase (G6PD) deficiency, the most common human genetic disorder. G6PD-deficient patients are at risk of life-threatening hemolysis when exposed to 8-aminoquinolines, the only class of drugs efficacious against *P. vivax *hypnozoites. Until recently, only qualitative tests were available in most settings. These can identify patients with severe G6PD deficiency (mostly male) but not patients with intermediate G6PD deficiency (always female). This has led to and reinforced a gap in awareness in clinical practice of the risks and implications of G6PD deficiency in females—who, unlike males, can have a heterozygous genotype for G6PD. Increasing recognition of the need for radical cure of *P. vivax*, first for patients’ health and then for malaria elimination, is driving the development of new point-of-care tests for G6PD deficiency and their accessibility to populations in low-resource settings. The availability of user-friendly, affordable, and accurate quantitative point-of-care diagnostics for the precise classification of the three G6PD phenotypes can reduce sex-linked disparities by ensuring safe and effective malaria treatment, providing opportunities to develop supportive counseling to enhance understanding of genetic test results, and improving the detection of all G6PD deficiency phenotypes in newborns and their family members.

Box 1. **Learning points**

1. Deficiency of the essential enzyme glucose-6-phosphate dehydrogenase (G6PD) in humans is caused by mutations in the *G6PD* gene located on the X-chromosome (Xq28). As such, males are either G6PD deficient or normal while females can be deficient, intermediate, or normal. The standard qualitative tests typically used to diagnose G6PD deficiency can identify deficient subjects but cannot reliably differentiate intermediate G6PD activity. Consequently, accurate assessment of G6PD status is more difficult in females, especially at the point of care where it is needed to inform *Plasmodium vivax* malaria case management.
2. Evidence for haemolysis associated with anti-malarials in G6PD-intermediate females has been documented, albeit infrequently, since 1958. Differentiating females with intermediate activity from those with normal activity has not been prioritised due to technical complexity of the testing. As a result, front-line health care providers are often unaware of the sex-related, oxidative drug–associated risk for female patients.
3. New quantitative point-of-care G6PD tests that provide accurate results for both males and females present important opportunities to address the sex-linked disparities related to safe and efficacious malaria treatment in women and girls. These tests can also be used to address other G6PD deficiency–associated medical indications, including improved management of neonatal hyperbilirubinemia in low-resource settings.
4. Realisation of these opportunities requires community engagement and improved counseling to enhance understanding of genetic test results and the implications for offspring and other family members.
5. Using G6PD testing beyond malaria treatment can further enhance the cost-effectiveness of the test, an important consideration for low-resource and malaria-endemic settings.

## Introduction

A new, single-dose radical cure for *Plasmodium vivax*, tafenoquine has received registration in Australia (Kozenis®), the United States (Krintafel®), and more recently in Brazil and Thailand. This combined with the drive to eliminate malaria, is encouraging malaria-endemic countries to increase access to testing for glucose-6-phosphate dehydrogenase (G6PD) deficiency. G6PD deficiency is the most common sex-linked genetic abnormality in humans, affecting more than 400 million people worldwide ^1, 2^, and it is associated with increased haemolytic risk during treatment with oxidative drugs, including radical curative treatments against *Plasmodium vivax* malaria. The World Health Organisation (WHO) estimates that there were 7.5 million cases of *P. vivax* in 2017 alone ^3^, a large proportion of which occurred in populations with high G6PD deficiency prevalence ^4^. For most countries outside sub-Saharan Africa approaching malaria elimination, *P. vivax* is now the main contributor to malaria disease burden, and there is recognition of the need for broader access to radical cure with either the current, standard, 7- to 14-day primaquine course or the new, single-dose tafenoquine, both of which are contraindicated in people with G6PD deficiency ^5– 7^. For G6PD-deficient patients with *P. vivax* malaria, WHO recommends an 8-week course of primaquine (0.75mg/kg weekly), which is qualified in the WHO malaria treatment guideline as a conditional recommendation with very low quality evidence. Where G6PD testing is not available, WHO recommends “all females should be considered as potentially having intermediate G6PD activity and given the 14-day regimen of primaquine, with counselling on how to recognise symptoms and signs of haemolytic anaemia” ^8^. Tafenoquine is the first drug to be contraindicated additionally in females with intermediate G6PD activity ^9– 13^.

**Figure 1.**
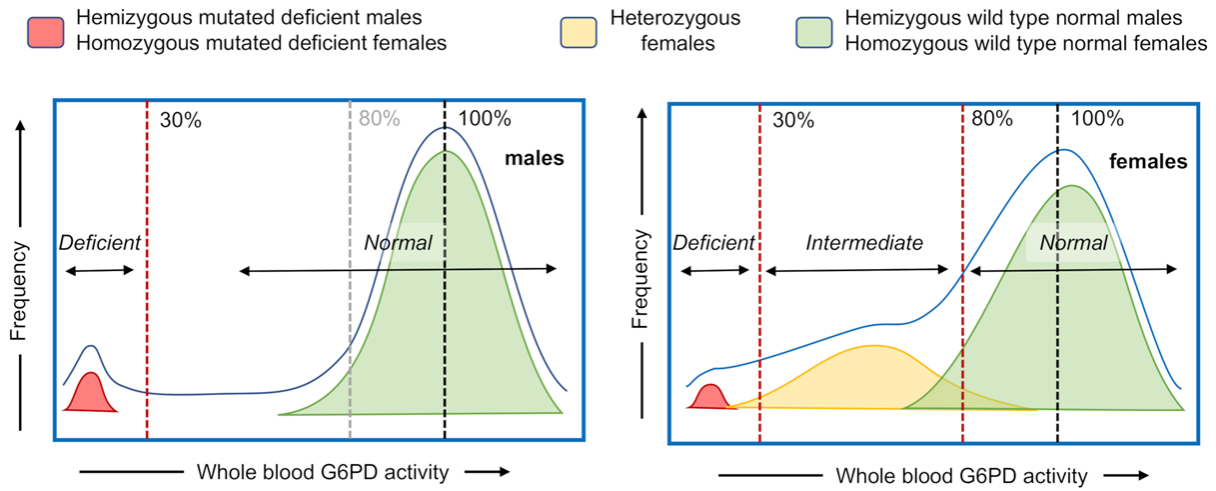
Schematic of population histograms demonstrating the relationship between phenotype and genotype in G6PD deficiency in males (left panel) and females (right panel). The *G6PD* gene is located on the X chromosome, such that females have two genes and males have only one. Males with a mutated *G6PD* allele (in red, G6PD _DEF_) that expresses a compromised (deficient) G6PD enzyme protein typically have a blood G6PD value of less than 30% of normal. Females with two mutated G6PD-deficient alleles (in red, G6PD _DEF1, DEF2_) also typically have a blood G6PD value of less than 30% of normal. Males with a wild type *G6PD* allele (in green, G6PD _WT_) that expresses a fully functional enzyme have G6PD activity in an approximate normal distribution around the 100% median, as do females with two wild type *G6PD* alleles (in green, G6PD _WT1, WT2_). Heterozygous females with both wild type and mutated *G6PD* alleles (in yellow, G6PD _WT, DEF1_) can express a spectrum of whole blood G6PD activity, ranging from severely deficient (<30%) to beyond the World Health Organization definition of normal for females (>80%), with the majority in the intermediate (30% to 80%) activity range. The colored zones indicate the distribution of enzymatic activities associated with the genotypes as described above; the blue line represents the cumulative G6PD activity-based histogram.

The *G6PD* gene is located on the X chromosome, so males have only one gene that expresses the G6PD enzyme and are either deficient in G6PD enzyme activity or normal, whereas females have two genes (but only one is expressed in each cell), and can have deficient, intermediate, or normal levels of G6PD activity ( Figure 1) ^2, 14^.

Historically, it has been challenging to accurately identify and diagnose females with intermediate G6PD deficiency because existing quantitative diagnostic tests are complex and require good laboratory infrastructure ^15– 17^. While qualitative rapid tests and other near-patient methods, such as the fluorescent spot test, may be sufficient to differentiate gross deficiencies, they do not reliably assess intermediate status. Instead, the tests misclassify females with intermediate enzyme activity as normal. As a result, males and females who are severely G6PD deficient can be identified correctly and treated with weekly primaquine (or no treatment in the case of other oxidative drugs), whereas females with an intermediate G6PD status remain under-diagnosed and thus inadvertently exposed to oxidative treatments.

As countries prepare to increase access to *P. vivax* radical cure with the curative regimens of primaquine ^18, 19^ and tafenoquine (Kozenis® and Krintafel®) ^11, 12^, respectively, there are several opportunities that should be considered within broader national health systems for expanding the acceptability, utility, and concurrently, the cost-effectiveness of G6PD testing beyond malaria case management ^15^. In this article, we discuss an important opportunity to reduce sex-related health disparities in an era of new tools and initiatives that improve the diagnosis of G6PD deficiency. This is in alignment with the need to recognise sex as a key determinant of health and its importance in health research to understand the impact on health outcomes ^20^. We also propose a research agenda to investigate this opportunity ( Table 1).

**Table 1.** Research questions to investigate the implications of a new drive to increase access to safe radical cure of *Plasmodium vivax* malaria, including diagnosis of intermediate G6PD deficiency in females and availability of new point-of-care tests for G6PD deficiency.

Research topics/agenda	Considerations
Studies to assess the feasibility of introducing point-of- care quantitative tests into health clinics in low-resource settings, including ethnographic/qualitative studies on a barriers/facilitators model.	A quantitative G6PD test may need to be integrated into dynamic and contextually specific malaria case management strategies, newborn screening policies, and other complex health services. Feasibility studies will help to ensure that this integration can be successfully scaled up across areas where G6PD deficiency is prevalent.
Ethnographic research to inform appropriate messaging and frontline staff training with respect to G6PD deficiency genetic counseling content and tools for target populations in low-resource, high disease burden settings.	Current practices vary widely and scant evidence exists regarding G6PD deficiency counseling best-practices. Ample lessons from other genetic conditions can be leveraged to design and validate such tools.
Studies comparing the costs of implementing current G6PD tests, including costs of inaccurate diagnosis of intermediate G6PD, and new point-of-care tests.	As new point-of-care tests become available, stakeholders within the health system will need clear guidance on the costs of various products. These costing studies will need to consider factors such as training, distribution, and product specifications such as shelf life.
Cost-effectiveness studies that take into consideration broader clinical benefits to the individual than those of *Plasmodium vivax* cure, in different G6PD prevalence settings.	Improve the value proposition of G6PD testing by considering other clinical benefits (not only as part of malaria case management) such as averting exposure to other oxidative agents, improved management of neonatal hyperbilirubinaemia in offspring and averting kernicterus.
Clinical studies to better define risk of clinical haemolysis in intermediate females given different anti-relapse regimens.	Clinical data focused on intermediate females will help inform downstream decisions regarding appropriate anti-relapse regimens. Currently, these decisions are often made based on a clinician’s individual risk-benefit assessments. The need for this type of medical expertise can restrict 8-aminoquinoline usage to the highest tiers of the health system.

### G6PD deficiency in females

In a given population with a mutated *G6PD* allele frequency of 10%, this same number will indicate the proportion of affected males. The proportion of females with the homozygous mutated genotype will be around 1%, while a large proportion of females (20%) will be heterozygous with a variable phenotype due to X-chromosome inactivation ^15, 18, 21^. Of the heterozygous females, around 60% will have an intermediate phenotype ^22^. Thus while males represent the highest proportion of individuals with severe G6PD phenotypes, there is also a comparable proportion of females with intermediate G6PD deficiency. While this is based on well-established knowledge, the characterisation of G6PD phenotypes in heterozygous women has received little attention at the patient level, possibly because of the more labor-intense laboratory techniques needed to characterise it ( Table 2). In clinical settings worldwide, the most commonly performed tests are qualitative tests (e.g., fluorescent spot test), which result in a systematic underestimation of the number of females at risk (as intermediates are classified as normal) and a perception among health workers that G6PD deficiency only concerns males ^23^.

**Table 2.** Scientific findings and technical advancement for the characterisation of women with intermediate G6PD activity.

Reference	Year published	Main findings
Beutler *et al*.	1955	Development of the GSH test for sensitivity to primaquine
Beutler *et al*.	1955	Development of the Heinz Bodies test for sensitivity to primaquine
Alving, *et al*.	1958	Primaquine associated haemoglobin reduction observed in individuals with intermediate activity
Childs, *et al*.	1958	
Gross, *et al*.	1958	
Tarlov, *et al*.	1962	
Brewer, *et al*.	1960 *	Development of the methaemoglobin reduction test (MRT) for sensitivity to primaquine
Stamatoyannopoulos, *et al*.	1967	Description of the enzymatic phenotypes in small samples of known G6PD heterozygous females
Panizon, *et al*.	1970	
Rinaldi, *et al*.	1976	
Beutler *et al*.	1977	Gold standard spectrophotometric assay
Van Noorden, *et al*.	1985	Description of new or improved cytochemical techniques for detection of G6PD in erythrocytes
Vives-Corrons, *et al*.	1986	
Vogels, *et al*.	1986	
Fanello, *et al*.	2008	Dapsone associated haemolysis in G6PD heterozygous females (no phenotype)
Premji, *et al*.	2009	
Tiono, *et al*.	2009	
Pamba, *et al*.	2012	
Shah, *et al*.	2012	Development of the cytofluorometric reading of MRT
Chu, *et al*.	2017	Primaquine and tafenoquine associated haemolysis in G6PD heterozygous females with intermediate activity
Rueangweerayut, *et al*.	2017	

* For ease of visualization, this article is listed out of chronological order

Other than the malaria radical curative indication, quantitative screening for G6PD deficiency can be expanded also geographically to clinical scenarios to improve patient care in females with intermediate G6PD status (where evidence is available) with an additional benefit of cost sharing:

(i) *Newborn screening to identify risk for pathologic hyperbilirubinaemia*. Evidence indicates that qualitative G6PD tests do not detect all clinically relevant cases of G6PD deficiency, particularly in female neonates ^24– 28^. This results in comparatively less emphasis on the clinical management of female neonates who have a risk of developing pathologic levels of serum bilirubin.
(ii) *The use of dapsone through the antimalarial drug chloroguanil-dapsone-artesunate (not used in malaria treatment anymore but is used for other medical indications).* Individual study analysis of phase 3 clinical trials with chloroguanil-dapsone-artesunate did not identify risk of severe haemolysis in G6PD heterozygous females ^29, 30^. However, a complete analysis of the data, including 200 heterozygous females, demonstrated that heterozygous females showed a wide range of reactions, from large to imperceptible haemoglobin drops ^31^. One published case report describes a Greek female who tested G6PD normal by a screening test then experienced severe haemolysis to dapsone; quantitative testing was recommended ^32^.
(iii) *Guidelines for rasburicase therapy in the context of genotyping*. The Clinical Pharmacogenetics Implementation Consortium published guidelines that recognise the limitations of *G6PD* genotype results to inform the use of rasburicase therapy in females for management of tumor lysis syndrome as *G6PD* genotyping does not correlate with the three phenotypes in heterozygous females ^33, 34^.

With the introduction of new quantitative point-of-care G6PD diagnostics, the three G6PD phenotypes can be detected accurately and characterised precisely for the first time without the need for expensive, complicated assays that require good laboratory infrastructure. Immediate results can be obtained at the patient level in the clinic. Quantitative G6PD testing could be used prior to prescription of other haemolytic agents (such as co-trimoxazole or nitrofurantoin) and new data could contribute to the literature on their effects in heterozygous females with intermediate G6PD activity. Where G6PD testing is driven by malaria case management, often rural and lower socioeconomic populations will benefit, as these populations often experience higher rates of malaria infection. Recent renewed attention to the characterisation at a cellular level of heterozygous G6PD deficiency may help to raise awareness of the clinical implications of intermediate G6PD activity in females ^35– 37^.

### *Plasmodium vivax* case management

A significant barrier to safe radical cure of *P. vivax* malaria with 8-aminoquinolines is the limited availability of G6PD tests. Until recently, primaquine has been administered either with no G6PD testing or with the use of qualitative tests ^38^. Females in the lower range of intermediate G6PD activity are at risk of potentially clinically significant haemolysis from high dose 14-day primaquine (0.5mg/kg daily) and tafenoquine (300mg single dose) (Kozenis® and Krintafel®) ^9, 13, 19, 39, 40^. In a systematic review comparing treatment with chloroquine versus chloroquine and primaquine, G6PD “normal” (classified using qualitative tests) females taking primaquine had significantly greater haemoglobin reductions than males ^41^. Recent studies exploring the efficacy of a high dose short course (7-day) primaquine regimen (1mg/kg) have shown consistently that females classified as normal by the fluorescent spot test are at risk for clinically significant primaquine-induced haemolysis ^19, 40^. One of the studies was carried out in an area with a high prevalence (15–18%) of G6PD deficiency; heterozygous females showed a significantly greater drop in haematocrit as compared to wild type homozygous females given the same treatments, and 2/16 females receiving dihydroartemisinin-piperaquine with the 7-day primaquine regimen needed a blood transfusion ^18, 40^. In the second study, 2 females from the site in Hanura, Indonesia had clinically significant haemolysis (a 31–40% haemoglobin reduction from pre-treatment) where 229 total participants were treated with the 7-day primaquine regimen ^19^. Prevalence of G6PD deficiency in the area was unknown but previous reports in West Timor region gave a prevalence of 3.2% among males ^42^. In clinical studies using tafenoquine 300mg (equivalent to the 14-day low dose primaquine regimen) where females with intermediate G6PD activity were excluded, no haemolysis-related adverse events were observed ^11, 12^. The inability to diagnose intermediate G6PD activity can negatively impact the safe radical cure of *P. vivax* malaria with high dose 14 or 7-day primaquine regimens and tafenoquine 300mg single dose in girls and women. The risk of haemolysis in malaria-endemic locations is concerning because of low access to medical supervision and health facilities where haemolytic events can be detected with haemoglobin testing and managed with blood transfusion.

From a policy and clinical practice perspective if national malaria programs decide to support radical cure with primaquine in the absence of G6PD testing, important ethical considerations will be raised and difficult tradeoffs between ensuring patient safety and expanding access to critical treatments must be made. In places where no testing is done, more males are at risk of severe haemolysis simply because there are more G6PD deficient males than females. In places where only qualitative testing is done, malaria programs may not be comfortable treating women given the limitations of that platform, as explained above. As such, some policies may indicate that radical cure be given only to males after qualitative G6PD testing at the point of care and all females referred to a health facility. In the absence of testing, practitioners may also assume all females (normal, intermediate and deficient) are G6PD deficient and they will be given the 8-week high dose primaquine regimen recommended for G6PD deficient individuals. These policies and practices generally are not standardized and can change quickly based on new information and varying perceptions of risk among key decision-makers. Decisions to include qualitative G6PD testing in clinical guidelines or use them as a matter of policy do provide broader access to G6PD testing and improve health disparities in malaria treatment. However, the benefit of this expanded access is mostly in males who have the most severe G6PD deficiency and are at highest risk of severe haemolysis. Alternatively, quantitative G6PD testing allows equal access to an accurate G6PD diagnosis, safe treatment and convenient health care delivery.

Anticipation of mandatory quantitative G6PD testing to support anti-relapse treatment of *P. vivax* with tafenoquine has spurred the development of new quantitative point-of-care G6PD diagnostic tests, which only recently have become commercially available. These tests demonstrate greater accuracy in identifying G6PD deficiency in females, including those with the intermediate phenotype ^43, 44^. A diagnosis of G6PD intermediate status presents an important opportunity to address disparities in appropriate treatment and care ^17^, such as providers’ low understanding and recognition of haemolytic responses, patients’ low awareness of adverse symptoms and the need for prompt follow-up, and appropriate genetic counseling.

### Increased newborn screening for G6PD deficiency

The diagnostic gap for G6PD testing extends to newborn screening globally. Newborn screening for G6PD deficiency has been recommended by the World Health Organization among populations where 3–5% of males are affected ^45^. It has been recognised that even if the majority of G6PD deficient patients are asymptomatic as children and adults, they have an increased risk of kernicterus resulting from significant neonatal hyperbilirubinaemia ^46^. Screening for G6PD deficiency is recommended in newborns with jaundice, especially when family history or background suggest the likelihood of G6PD deficiency, or when the response to phototherapy is poor ^45, 47– 50^. Nonetheless, there is a high heterogeneity in practice among different countries and within countries between rural and urban areas, with urban and peri-urban areas having greater access to G6PD screening programs. The same heterogeneity applies to screening methods, with G6PD deficiency screening performed in some settings via high accuracy, gold-standard quantitative spectrophotometric methods, while in low-resource settings it is more commonly done using low-accuracy, qualitative diagnostic tests.

The diagnostic limitations of qualitative tests restrict the potential for downstream public health interventions to improve clinical care for all infants, particularly female infants. For example, a robust G6PD newborn screening program paired with health education programs implemented in Sassari District, Sardinia, Italy, resulted in a 75% reduction in G6PD deficiency–related complications, showing that individual diagnosis helped prevent haemolytic triggers in the at-risk population of young male children. The benefit was observed disproportionally in boys, suggesting the intervention had been less effective in girls, in part because girls with low-intermediate G6PD activity were misclassified as normal and not “at risk.” ^51^.

Most point-of-care tests for G6PD deficiency currently are not indicated for use with neonates and clinical studies to support this indication should be performed. Quantitative point-of-care G6PD tests should enable diagnosis of female newborns with the intermediate phenotype. This could improve management of neonatal hyperbilirubinaemia, including closer clinical follow-up with targeted early bilirubin testing, avoidance of haemolytic triggers, and focused parental support on signs and symptoms of hyperbilirubinaemia to prevent kernicterus ^26– 28, 52^. Additional studies using new quantitative point-of-care tests for G6PD deficiency in the first year of life will show whether it is possible to use the results obtained at birth to provide a definitive diagnosis of the phenotype at least as G6PD deficient, or normal with intermediate perhaps requiring further follow up. This, coupled with the capacity to retain data throughout a patient’s life via personal or health system records, may enable once-per-lifetime testing of G6PD status.

As with adult management of malaria, newborn screening with only qualitative G6PD tests will identify males who are most frequently at the highest risk of developing G6PD related complications in the early neonatal period but will miss G6PD intermediate females. This means that largely avoidable G6PD related complications in female infants may be managed insufficiently, again introducing health related gender disparities from birth ^26– 28, 52^. Using quantitative G6PD testing for newborn screening would eliminate this health disparity.

### Counseling with G6PD testing

While G6PD deficiency testing has been conducted systematically in certain settings to support malaria treatment, it is not usually treated as a test requiring genetic counseling. Little guidance currently exists to help health care workers relay results to patients, explain the hereditary nature and X-linked inheritance pattern of the condition, and describe the clinical implications. While genetic counseling is still emerging in its application in low-resource settings ^53– 55^, the malaria field could draw lessons from genetic counseling in other domains, such as sickle cell disease, thalassaemia, other haemoglobinopathies, prenatal testing, and cancer genetics ^56– 61^. In many regions, where malaria is highly prevalent, inherited blood disorders overlap in prevalence, such that investment in genetic counseling capacity-building in these areas may be leveraged ^22^. This could be achieved by increasing the number of genetics professionals being trained annually and building such opportunities into genomics research projects, or by equipping other healthcare staff, including nurses and community healthcare workers to interpret genetic knowledge ^62^.

While an individual can potentially go through life unaware of his or her G6PD deficiency, there are still lifelong benefits to being informed. Particularly for women, awareness of G6PD status and understanding of inheritance bring a direct clinical advantage, with the added benefit of prompting testing in their newborns and other family members. In low-resource settings, screening for haemoglobinopathies often occurs too late to intervene, either during a pregnancy or after the birth of the severely sick child, bringing social stigma to the woman who “caused” the disease in the child ^63^. In contrast, for G6PD deficiency, an opportunity exists to develop genetic counseling specifically designed to minimise stigma and maximise the importance of knowing one’s G6PD status in order to prevent haemolytic triggers actively and inform other decisions throughout an individual’s lifespan, such as food and environmental factors to avoid. Counseling may be particularly important to clarify potential gender biases given the X-linked inheritance and variable penetrance amongst male and female individuals, as well as population biases due to increased screening of certain populations ^64^.

### Community engagement and sensitisation

Community engagement is essential for creating awareness and minimising stigma ^63^. Extensive literature indicates the importance of community engagement and community leadership for the success of programs involving screening for genetic disorders ^56, 59, 61, 65, 66^, including some targeting G6PD deficiency in Sardinia, Italy ^51^. Lessons from studies performed at the community level for neglected tropical diseases suggest that coverage rates, adherence rates, and general acceptability of new interventions improve when communities and community leadership are involved ^67, 68^. For example, in a recent evaluation of rapid tests for onchocerciasis, participants reported that the manner in which new tests were introduced and results were provided influenced community perceptions of the acceptability of the tests and confidence in the test results ^69^.

These same strategies can be adapted and applied to the expansion of G6PD diagnostics. Women who learn their G6PD status—and who understand the broader consequences of their condition through enhanced genetic counseling—may encourage their family members to get tested. This could have an important ripple effect within families and communities in malaria-endemic settings, whereby more individuals seek testing (possibly independent of a malaria episode), learn their G6PD status, and use this information for decisions beyond malaria treatment.

### Cost-effectiveness of G6PD testing

The health and economic advantages of introducing quantitative, point-of-care G6PD testing in malaria case management will be optimised if the clinical benefits of testing can be extended beyond the current primary indication, which is for malaria treatment. At a minimum, retention of the G6PD test result by the patient or the health system may eliminate the need to test again the next time a patient requires radical cure for *P. vivax* or develops a haemolytic crisis, during which testing would not be reliable ^70^. At a higher level, knowing one’s G6PD status at subsequent clinical visits would allow the patient and clinician to prevent a severe haemolytic crisis by avoiding oxidative medications, or if such a prescription is unavoidable, to closely monitor for signs of haemolysis so interventions could be performed earlier. This benefit could expand even further if, through appropriate community sensitisation and genetic counseling, a woman with intermediate G6PD activity seeks G6PD testing for her newborn. This could prompt closer follow-up care and avert hyperbilirubinaemia-related complications. For example, in Singapore, the implementation of universal screening for G6PD deficiency in the context of the Kernicterus Surveillance Programme has led to elimination of kernicterus in the country ^71, 72^, which is cost effective at the household and national level.

Clearly, when G6PD status can be retained by the individual or medical facility, repeated testing is not necessary, which in turn generates cost savings. However, when G6PD status cannot be reliably retained (e.g., in migratory populations) or requires confirmatory testing (i.e., initial test during an acute illness), repeated G6PD testing will reduce the cost-effectiveness of any health-related program, such as malaria elimination.

Current rough estimates indicate that a quantitative reader machine for G6PD (i.e., biosensor) will be in the range of 50–300USD, with test kits in the range of 3–5USD per unit; this will likely be less expensive than gold-standard spectrophotometric assay in terms of equipment (reader vs. temperature-controlled spectrophotometer), reagents (temperature-stable and ready-to-use vs. refrigerated), and operational time and time to result (clinical staff vs. specialised laboratory), but more expensive than the widely used qualitative fluorescent spot test or RDT. If only a G6PD qualitative RDT (1.75 USD per test) is used, the estimated cost per person over one year per episode is 39.10 USD in Thailand (Devine *et al.*, 2017). However, the household and provider cost of one *P. vivax* malaria episode to an individual in the same country is estimated to be 150 USD (Devine *et al.*, 2019).

Point-of-care quantitative tests could be used in secondary-level health care facilities. Introduction in selected primary-level facilities would depend on the ease of use by health care providers who deliver care at this level and is a considerable limitation to the implementation of quantitative G6PD testing in field settings. Depending on country settings, health seeking behaviors and healthcare access, a significant proportion of malaria patients would still not have access to safe radical cure unless radical cure can be administered safely by village health workers. Alternative service delivery models would need to be investigated to extend the reach of 8-aminoquinolines while ensuring safety. A combination of screening males with the more affordable qualitative test and females with the quantitative test may reduce commodity costs, but operational feasibility and costs of maintaining two products and training on their appropriate would need to be investigated.

With increased utilisation of and experience in quantitative G6PD testing, data will need to be captured to estimate precisely the associated costs and benefits across all performance domains. This will better inform future test development. It will also impact country-level decision-making in terms of cost trade-offs within limited health care budgets and acceptable costs for incorporating G6PD testing into health systems. The cost-effectiveness will be weighted in part by the prevalence of G6PD deficiency in a population. For example, if the prevalence is low or negligible, the benefits of testing for G6PD deficiency may be limited to the clinical benefit for which it was indicated, most likely radical cure of *P. vivax* malaria ^73– 75^. Because efficacy of radical cure is highly related to completion of primaquine regimens, ensuring dose adherence will greatly enhance the cost effectiveness; this is less of a challenge with the single day tafenoquine regimen. Deliberations over cost-benefit trade-offs at the country and district level should also include careful consideration of the ethical and equity considerations discussed above.

## Conclusion

The sex-linked differences in G6PD deficiency and limitations of current G6PD diagnostic tools have led to a disparity in accurate G6PD diagnosis in females. The interaction of *P. vivax* malaria, G6PD status, 8-aminoquinolines, and treatment restrictions linked to pregnancy and the post-partum period results in a sex related inequity. Females are at higher risk for misclassification of a sex-linked disorder in women, low awareness of the risk for iatrogenic haemolysis of the G6PD intermediate phenotype, and no *P. vivax* anti-relapse vivax treatment for a large part of their reproductive life. 

Standard high-dose primaquine and new anti-relapse treatment regimens against *P. vivax* malaria require G6PD testing. New, affordable, point-of-care quantitative G6PD diagnostics have been developed to support access to these drugs in malaria-endemic populations. In addition to the benefits at multiple levels of the health care system, these new diagnostic tools can help bring awareness to the front-line practitioner of the nuances of G6PD deficiency in females and the potential implications beyond malaria.

Furthermore, integration of G6PD test results across multiple clinical indications, such as hyperbilirubinaemia in neonates, will likely require improved genetic counseling, health systems strengthening, and improved record keeping and data management. In settings in which G6PD deficiency is prevalent, these efforts may result in greater cost-benefits beyond the use of G6PD tests for malaria treatment alone, and lead to more equitable malaria treatment.

## Data availability

### Underlying data

No data are associated with this article
