# Supplementary material for: Optimizing G6PD testing for Plasmodium vivax case management and beyond: why sex, counseling, and community engagement matter
Source: Wellcome Open Res. 2020 Aug 25;5:21. Originally published 2020 Feb 7. [Version 2] doi: 10.12688/wellcomeopenres.15700.2 (PMC7388194; doi:10.12688/wellcomeopenres.15700.2)
Supplement: Peer review history

# Peer review: Reviewer response for version 1

**DOI:** 10.21956/wellcomeopenres.17206.r38264

**Published:** 2020-07-28

**Authors:** Manoj Menon

**Affiliations:** 1 Clinical Research Division, Fred Hutchinson Cancer Research Center, Seattle, WA, USA

In this review, "Optimizing G6PD testing for Plasmodium vivax case management: why sex, counseling, and community engagement matter", the authors advocate for the need for quantitative testing for G6PD - which could help identify an intermediate phenotype and potentially at risk of the sequelae of hemolysis. This would help foster both a clinical benefit as well as improve health equity.

1. Would consider specifying the danger of G6PD deficiency (as well as G6PD intermediate) earlier in the introduction.
2. Introduction, paragraph 1: "For most countries approaching malaria elimination *, P. vivax* is now the main contributor to malaria disease burden," Is this for coendemic countries or all countries? Please specify and source.
3. Introduction, paragraph 3: What percentage of intermediate test results are misclassified as normal?
4. Given that this review is meant to focus on G6PD testing for *P. vivax*, I wonder if there is too much emphasis on the newborn screening. It is obviously an important component, but not as related to the topic (as per the title of the review article)
5. "Guidelines for rasburicase therapy in the context of genotyping". This seems a bit out of place, the use of rasburicase for the management of tumor lysis syndrome (a relatively rare event) would be so uncommon in areas of malaria endemicity. There are other drugs which, I think, are more common: e.g. Co-trimoxazole, nitrofurantoin, possibly aspirin)
6. Counseling with G6PD testing, paragraph 1, 2nd sentence. Would clarify that G6PD is inherited in an x-linked pattern, not an "autosomal dominant inheritance pattern"
7. With regard to cost-effectiveness, would it be an effective strategy to use the less expensive qualitative test for males and the quantitative test for females?
8. The figure (population historgram) and table 1 (research agenda) are helpful. Consider discussing in the text.

Thank you for allowing me to review this interesting manuscript.

Is the review written in accessible language?

Yes

Are all factual statements correct and adequately supported by citations?

Yes

Are the conclusions drawn appropriate in the context of the current research literature?

Yes

Is the topic of the review discussed comprehensively in the context of the current literature?

Yes

Reviewer Expertise:

hematology, (previously malaria)

I confirm that I have read this submission and believe that I have an appropriate level of expertise to confirm that it is of an acceptable scientific standard.

# Response

**Published:** 2020-08-06

**Authors:** Cindy Chu

**Affiliations:** Faculty of Tropical Medicine, Shoklo Malaria Research Unit, Mahidol University, Thailand

We would like to thank Reviewer 2 for their helpful comments on this manuscript.

1. **Reply:** To improve the rationale of this paper earlier in the introduction, we have modified the 2 ^nd^ sentence of the introduction to read “This combined with the drive to eliminate malaria, is encouraging malaria-endemic countries to increase access to testing for glucose-6-phosphate dehydrogenase (G6PD) deficiency. *G6PD deficiency is *the most common sex-linked genetic abnormality in humans, affecting more than 400 million people worldwide, *and it is associated with increased haemolytic risk during treatment with oxidative drugs, including radical curative treatments against Plasmodium vivax malaria*.”
2. **Reply:**  We have specified the sentence as follows “For most countries *outside sub-Saharan Africa* approaching malaria elimination, *P. vivax* is now the main contributor to malaria disease burden….”, and a reference to the WHO malaria report, 2019 has been added.
3. **Reply:** Qualitative tests are not built to identify intermediate levels of G6PD activity, therefore when using qualitative tests, 100% of females with intermediate activity are classified as normal. The fluorescent spot test is sometimes considered as a semi-quantitative test but in the authors’ experience the capacity to identify intermediate activities with this test is very limited.
4. **Reply:**  We agree with the reviewer and we have now modified the title in order to capture the different components of the manuscript. In relation to the newborn screening, we think that using G6PD testing more broadly (both within malaria endemic countries and outside for neonatal screening) can enhance the cost-effectiveness of the test and possibly even lower its price at the benefit of low-resource and malaria-endemic settings.
5. **Reply:** This section was written to provide additional clinical scenarios (with data on G6PD heterozygous females with intermediate G6PD deficiency) where quantitative G6PD testing could be used to benefit females with an intermediate G6PD status; linking to the section on ‘Cost effectiveness’, later in the paper. As the reviewer states, there are other more commonly used drugs where quantitative G6PD testing would be helpful however, data on heterozygous intermediate G6PD females are available only in the clinical situations as stated in the paper (rasburicase, dapsone, neonatal hyperbilirubinaemia). To clarify we have amended the sentence in the second paragraph of the section ‘G6PD deficiency in females’ to “Other than the malaria radical curative indication, quantitative screening for G6PD deficiency *can be expanded to *clinical *scenarios to improve patient care in females with intermediate G6PD status (where evidence is available) with an additional benefit of cost sharing. ”  *We have also added a sentence at the end of the section: “ *Quantitative G6PD testing could be used prior to prescription of other haemolytic agents (such as co-trimoxazole or nitrofurantoin) as more data are collected on their effects in heterozygous females with intermediate G6PD activity*.”
6. **Reply:** We have corrected this to ‘X-linked’. Thank you for noticing this error.
7. **Reply:** This would certainly reduce the cost per test from 2-3 USD for a G6PD Biosensor test compared to 1-2 USD for the Carestart G6PD RDT. It may not improve the cost effectiveness of the quantitative G6PD testing device, which becomes more cost effective if it is used frequently. There are also added importation fees in some countries that would affect the cost effectiveness. Ideally, each country, hospital, or region would decide on what is cost effective for their local context. We have inserted the following sentence in the cost effectiveness section: *“* *A combination of screening males with the more affordable qualitative test and females with the quantitative test may reduce commodity costs, but operational feasibility and costs of maintaining two products and training on their appropriate would need to be investigated.”*
8. **Reply:**  We appreciate the reviewer’s comments, but given already the extensive length of the article, we would like to keep the description of the Figure 1 in the figure legend and research agenda/topics contained in the table. We hope this is acceptable to the reviewer.

# Peer review: Reviewer response for version 1

**DOI:** 10.21956/wellcomeopenres.17206.r39288

**Published:** 2020-07-15

**Authors:** Chansuda Wongsrichanalai

**Affiliations:** 1 Independent Consultant, Bangkok, Thailand

This review paper addresses the issues of access to effective radical treatment of *P. vivax* malaria. The danger of hemolysis associated with the use of 8-aminoquinoline drugs in G6PD-deficient malaria patients is emphasized. The authors provide an excellent review of the genetic basis of G6PD deficiency and the limitation of tools in the past to detect G6PD enzyme at Point-of-Care (PoC).

 The recently-available PoC quantitative G6PD test is pointed out as an innovative tool that improves safe *P. vivax* radical cure by reporting the enzyme levels, which help to guide more precise 8-aminoquinoline treatment choices. As such, it offers safer primaquine treatment for heterozygous females. Additional benefit of the PoC quantitative G6PD tests for newborn screening to determine the risks of hyperbilirubinemia is also clearly discussed and possible research opportunities are suggested.

**Comments:**

1. In the Abstract “….. the availability of SIMPLE, affordable….”
  By default, most readers working on malaria in the field would think of a SIMPLE test being somewhat similarly simple to malaria rapid test (RDT). Because the current PoC quantitative G6PD test is still not that simple, this sentence should be modified. The issue of test simplicity is considered by many field malaria workers as its constraint; this should also be mentioned somewhere in the paper. Probably the next generation of the test could be made simpler for users in public health services in the heart of remote malaria endemic areas?
  Deployment of the current PoC quantitative G6PD tests will have limitations in some malaria settings depending on the levels of health/economic development of the country. The authors touch lightly on this matter (3 ^rd^ paragraph of Cost-Effectiveness) indicating that the test “could be used in secondary-level health care facilities and introduced in selected primary-level facilities” but in practice it can be much more complicated. For example, in Cambodia, the test is not yet user-friendly enough to be operable by Village Malaria Workers or at many rural health centers (where a large percentage of malaria cases are detected). The national malaria statistics would show the number of patients served by these different health care levels (with different lab facilities/personnel capability), so it might be of interest to determine what percentage of *P. vivax* patients could potentially benefit from this new G6PD testing in such a country.
2. Cost-effectiveness
  The authors made some good suggestions to apply PoC quantitative G6PD test beyond malaria to improve cost-effectiveness. However, to consider cost-effectiveness of *P*. vivax malaria treatment specifically, it would be useful to take into consideration parallel efforts to improve adherence to multi-days primaquine regimen. As failure to complete the required primaquine doses is known to be a key obstacle to *P. vivax* radical cure, detection of G6PD deficiency and the ability to prescribe primaquine safely alone would not be sufficient to contribute to treatment effectiveness; we need to enhance compliance to therapy.
3. Neonatal screening for the risk of hyperbilirubinemia
  The authors should make it clear to the readers that this is not an indication of the current G6PD test products (as far as this reviewer has learnt). If any test that is designed for that indication is already available, please specify. The review of using the test for screening at birth is very informative but there is a chance of readers being misled to assume that the products available in the market now are meant for that purpose. Do we still need clinical studies to assess such an off-label use (i.e. for neonatal screening)? Or a new PoC product intended for newborn G6PD screening is being developed?
4. Under *Introduction*, the top line
  If possible the text should be updated so readers will be aware that tafenoquine has also been registered in *P. vivax* endemic countries such as Brazil and Thailand.

Is the review written in accessible language?

Yes

Are all factual statements correct and adequately supported by citations?

Yes

Are the conclusions drawn appropriate in the context of the current research literature?

Partly

Is the topic of the review discussed comprehensively in the context of the current literature?

Yes

Reviewer Expertise:

malaria epidemiology, drug-resistant malaria, malaria diagnosis

I confirm that I have read this submission and believe that I have an appropriate level of expertise to confirm that it is of an acceptable scientific standard.

# Response

**Published:** 2020-08-06

**Authors:** Cindy Chu

**Affiliations:** Faculty of Tropical Medicine, Shoklo Malaria Research Unit, Mahidol University, Thailand

We would like to thank Reviewer 1 for their constructive comments on this manuscript.

1. **Reply:** In the abstract, we have changed the word “simple” to “ *user-friendly*”. The issue of test simplicity is addressed in the 3 ^rd^ paragraph of the Cost effectiveness section. We have amended the last sentence to read “ *Introduction* in selected primary-level facilities *would depend on the ease of use by health care providers who deliver care at this level and is a considerable limitation to the implementation of quantitative G6PD testing in field settings*.”  We agree with the reviewer that getting the radical cure only to primary health care facilities may significantly curtail the percentage of patients who benefit.  To reflect this, we have inserted into the ‘Cost effectiveness of G6PD testing’ section the sentence below. There are reasons beyond that of the complexity of G6PD testing (for example complexity of treatment algorithm) that may impose the need to consider alternative service delivery models. “ *Depending on country settings and health seeking behaviors, a significant proportion of malaria patients would still not have access to safe radical cure unless radical cure can be administered safely by village health workers. Alternative service delivery models would need to be investigated to extend the reach of 8-aminoquinolines will ensuring safety*”.We agree with the reviewer that getting the radical cure only to primary health care facilities may significantly curtail the percentage of patients who benefit.  To reflect this, we have inserted into the ‘Cost effectiveness of G6PD testing’ section the sentence below. There are reasons beyond that of the complexity of G6PD testing (for example complexity of treatment algorithm) that may impose the need to consider alternative service delivery models. “ *Depending on country settings and health seeking behaviors, a significant proportion of malaria patients would still not have access to safe radical cure unless radical cure can be administered safely by village health workers. Alternative service delivery models would need to be investigated to extend the reach of 8-aminoquinolines will ensuring safety*”.
2. **Reply: **This is a good point. We have introduced the following statement: *“Because efficacy of radical cure is highly related to completion of primaquine regimens, ensuring dose adherence will greatly enhance the cost effectiveness; this is less of a challenge with the single day regimen Kozenis. “*
3. **Reply:** It is true that the messaging that accompanies a new test should be clear and consistent to avoid misunderstanding by the user. From the authors’ perspective a G6PD quantitative test could be used in any situation where a diagnosis of deficient, intermediate or normal G6PD deficiency would improve clinical management. For newborns the thresholds for defining these categories may be different (likely higher). While there is one near-patient instrument: Finder from Baebies,  ( https://baebies.com/products/finder/) that seems to be indicated for this purpose, it is unlikely to be suitable for low resource settings. In agreement with the reviewers comment we have introduced the following statement: “ *Most point-of-care tests for G6PD deficiency are not currently indicated for use with neonates. Clinical studies to support this indication should be performed.”*
4. **Reply:** We have amended the sentence to include the Brazil and Thailand as countries that have registered tafenoquine. “A new, single-dose radical cure for *Plasmodium vivax*, tafenoquine has received registration in Australia (Kozenis®), the United States (Krintafel®), *and more recently in Brazil and Thailand.*”
